# Distinguishing Between Models for Extreme and Midpoint Response Styles as Opposite Poles of a Single Dimension versus Two Separate Dimensions: A Simulation Study

**DOI:** 10.1177/01466216251379471

**Published:** 2025-09-13

**Authors:** Martijn Schoenmakers, Maria Bolsinova, Jesper Tijmstra

**Affiliations:** 1 Department of Methodology and Statistics, 120694 Tilburg University, Tilburg, Netherlands

**Keywords:** response styles, extreme response style, midpoint response style, item response theory, multidimensional nominal response model, simulation study

## Abstract

Extreme and midpoint response styles have frequently been found to decrease the validity of Likert-type questionnaire results. Different approaches for modelling extreme and midpoint responding have been proposed in the literature, with some advocating for a unidimensional conceptualization of the response styles as opposite poles, and others modelling them as separate dimensions. How these response styles are modelled influences the estimation complexity, parameter estimates, and detection of and correction for response styles in IRT models. For these reasons, we examine if it is possible to empirically distinguish between extreme and midpoint responding as two separate dimensions versus two opposite sides of a single dimension. The various conceptualizations are modelled using the multidimensional nominal response model, with the AIC and BIC being used to distinguish between the competing models in a simulation study and an empirical example. Results indicate good performance of both information criteria given sufficient sample size, test length, and response style strength. The BIC outperformed the AIC in cases where no response styles were present, while the AIC outperformed the BIC in cases where multiple response style dimensions were present. Implications of the results for practice are discussed.

## Introduction

The use of Likert scales (e.g., answer scales from 1 to 5) in questionnaires throughout social science is widespread (Croasmun & Ostrom, 2011; Sullivan & Artino, 2013; Van Vaerenbergh & Thomas, 2013). It is, however, uncertain whether all participants utilize Likert response options in the same manner (Plieninger & Meiser, 2014). For example, some people may prefer to answer questions using only the ends of the scale, while others tend to answer using the middle response option. These tendencies of participants to answer items a certain way regardless of content are referred to as response styles (Plieninger & Meiser, 2014; Van Vaerenbergh & Thomas, 2013). A variety of response styles exist, for example, tending to use endpoints of the scale (extreme response style; ERS), a tendency towards the midpoint of the scale (midpoint response style; MRS), a tendency towards agreeing with items (acquiescent response style; ARS), and many more.

Participants exhibiting these response styles have repeatedly been found to reduce the validity of results obtained from Likert questionnaires in two ways (Van Vaerenbergh & Thomas, 2013). First, means and variances of latent variable scores may be affected. For example, participants displaying ARS may cause scale means to increase (for positively worded items) and scale variances to decrease. Previous research also noted the potential of MRS to bias scores towards the midpoint of the scale, particularly in cases where the true score is far from the middle of the scale (Baumgartner & Steenkamp, 2001). Second, and perhaps more importantly, systematic error can be introduced into results if some participant groups differ in their response style (Schoenmakers et al., 2023). For instance, a study by Moors (2012) found that a higher amount of ERS in women than in men led to a spurious relation between gender and leadership styles. It is thus important to detect and correct for response styles to prevent unwarranted conclusions from being drawn. As ERS and MRS are two often studied response styles, we discuss them in more detail below.

ERS has received much attention as a possible source of bias in questionnaire data (Greenleaf, 1992; Hui & Triandis, 1989; Morren et al., 2011; Naemi et al., 2009; Wetzel et al., 2013). Results may be biased by ERS in two important ways. First, ERS may result in bias concerning measurement of the latent variable and its relation to other variables (Batchelor et al., 2013) as exemplified earlier by the spurious correlation between gender and leadership styles (Moors, 2012). Second, the addition of construct-irrelevant variance can also reduce the magnitude of estimated associations between constructs, analogous to the attenuation of correlations that occurs when introducing random measurement error. For example, one study found that ERS reduced the explained variance from 69.5% to 53.5% (Lau, 2007). Note that ERS can thus both lead to spurious correlations as found in Moors (2012), and to attenuation of correlations as found in Lau (2007).

Due to the various negative effects of ERS on questionnaire results, many studies have attempted to link ERS to various person characteristics, such as personality (Naemi et al., 2009), culture (Hui & Triandis, 1989), race, gender, education, and intelligence (Batchelor et al., 2013). In addition, properties of the questionnaire have been found to affect ERS, such as response format (Lau, 2007), and remarkably even the visual distance between response options and whether the questionnaire options were presented vertically or horizontally (Weijters et al., 2021).

While much research has been done on the predictors of extreme responding, the question of how well extreme responding can be detected in a dataset remains relatively unanswered. Simulation studies done on extreme responding instead tend to focus more on the impact of extreme responding on outcomes and correcting for the extreme response styles (Plieninger, 2017; Wetzel et al., 2016, for examples). One notable exception is a study by Jin and Wang (2014), which introduced a new model for ERS. In addition to this primary aim, they briefly examined the accuracy of the DIC for detecting ERS as a non-primary research question. While this study offers encouraging results in establishing the presence of ERS compared to a model with no response styles, the DIC can only be utilized in Bayesian applications (Jin & Wang, 2014). The paper also does not examine the possibility of distinguishing ERS and MRS as a single dimension from ERS and MRS conceptualized as two separate dimensions, limiting itself to comparing a model with ERS to a model with no response styles.

Just as ERS, MRS has received much attention as a possible source of bias. MRS may result in invalid measurement of participants, and lead to artificially lowered or heightened correlations between variables (Van Vaerenbergh & Thomas, 2013). The effect of MRS on the variance is, however, quite different from ERS. Where ERS generally adds construct irrelevant variance, MRS generally deflates variance by making participants more often choose the midpoint of the scale. The deflated variance can lead to an increased magnitude of estimated associations between variables, increasing the risk of spurious findings (Van Vaerenbergh & Thomas, 2013).

Several studies have attempted to explain causes for MRS. Person characteristics that have been linked to MRS include evasiveness (not wanting to reveal one’s true opinion), indecision, and indifference (Baumgartner & Steenkamp, 2001). In addition, some research has established the relation between MRS and socioeconomic development, religious denomination, Hofstede values, Schwartz values and traditionalism at the country level (He et al., 2014). Again, while research has explored the impact of MRS on obtained results, and the causes of MRS have been studied, the detection of MRS has received surprisingly little research.

While the influences of ERS and MRS response styles on questionnaire data are relatively clear, the relation of ERS and MRS to each other is not. ERS and MRS are sometimes modelled as opposite ends of a single dimension, and other times modelled as two separate, independent dimensions (Falk & Cai, 2016). The decision to model ERS and MRS as two separate versus one singular dimension has various consequences. First of all, this modelling choice will result in a more or less complex model, with different item parameters and different estimated values for the latent trait of interest. Second, the precision of the estimates for the latent trait of interest may be affected. Third, the ease of estimating the response style model may be influenced. Fourth, the conceptual basis of the model shifts if we assume ERS and MRS are opposite poles of a single dimension as opposed to separate dimensions. This also has consequences for how we conceptually explain the presence or lack of the response style. Finally, the correction for the response style will take a different form depending on which operationalization is used.

For the aforementioned reasons, we will examine the following research question: Is it possible to empirically distinguish extreme responding and midpoint responding as separate dimensions or as opposite poles on a single dimension, and if so, under which conditions and with what degree of error? Note that we thus do not examine whether ERS and MRS are conceptually separate dimensions or not, but instead examine whether we can distinguish between ERS and MRS as separate dimensions or opposite poles of one dimension in given datasets. In order to answer this question, a simulation study will be conducted. The paper will proceed as follows: first, we discuss various approaches to modelling response styles and justify our chosen approach in the “Modelling Response Styles” section. Second, we will provide details on the methods of this study and the conditions chosen for the simulation in the “Methods” section. Third, the results are displayed in the “Results” section, and finally the “Discussion” section contains a discussion of the results and recommendations for practice and future research.

## Modelling Response Styles

A variety of approaches to modelling, measuring, and correcting for response styles exists. One of the most notable differences between these approaches is the conceptualization of response styles as categorical or continuous traits. When conceptualizing ERS as a categorical trait, mixture IRT models can be utilized. These models create several latent classes based on observed responses, where person and item parameters are estimated separately for each class (Rost, 1991). While classes can thus differ in parameters, within-class homogeneity is assumed. This lack of within-class variation is often not theoretically justified, as individuals within a class may very well differ in the presence and strength of their response style tendencies (Huang, 2016). As categorical traits are not able to model this within-class variation, the present paper will focus on models conceptualizing response styles as continuous traits.

Various models conceptualizing response styles as continuous traits have been developed. Examples include rating scale, partial credit, generalized partial credit, graded response models, and unfolding models (Javaras & Ripley, 2007; Jin & Wang, 2014; Johnson, 2003; Wang et al., 2006; Wang & Wu, 2011). In addition, IRTree models have been utilized for modelling various response styles (Bockenholt, 2012; Bockenholt & Meiser, 2017; Schoenmakers et al., 2023; Thissen-Roe & Thissen, 2013). Finally, various approaches using the multidimensional nominal response model exist (Bolt & Newton, 2011; Falk & Cai, 2016).

While the IRTree models offer a great deal of flexibility in modelling response styles, several problems prevent their use in our study. First, the recoding of data into nodes corresponding to a tree diagram (where many different tree diagrams and tree node operationalizations are possible, which are likely to result in different outcomes) complicates use of these models.

Second, the lack of a standard implementation in software of the more recent multidimensional node IRTree parameterizations (Alagöz & Meiser, 2023; Meiser et al., 2019) can reduce the ease of implementation of these models in practice. Multidimensional nominal response models can more easily be implemented using existing R packages (see e.g., Falk & Ju, 2020) and are thus more likely to be utilized by applied researchers.

Third, traditional operationalizations of IRTree models make an extreme response conditional on a non-midpoint response in the first node (Bockenholt, 2012; Plieninger, 2017), or a midpoint response conditional on a non-extreme response in the first node (Plieninger, 2017). In the first case, this means that no information regarding a person’s extreme response tendency or the substantive trait is given in the case of a middle response. In the second case, this means no information regarding a person’s midpoint response tendency or the substantive trait is given in the case of an extreme response. Both cases result in a reduced amount of information for one of the response styles and the substantive trait compared to the MNRM.

Fourth, and most importantly, the operationalization of ERS and MRS as opposite poles of a single dimension is conceptually problematic in IRTree models such as the ones described above. That is, these models fundamentally have to treat choosing the middle response category as a nonresponse choice rather than selecting a valid informative position located ordinally between the disagreeing and agreeing options. As a consequence, the latent variable in the node that captures this middle response category choice has to capture a nonresponse tendency (Tijmstra & Bolsinova, 2025; Tijmstra et al., 2018) rather than a tendency for informative responses to be shifted towards the center of the scale (as it does in the MNRM operationalization). This is in fundamental contrast to the ERS dimension in IRTree models, which does not capture a nonresponse tendency but instead affects the strength of the informative response option that is chosen. Thus, considering an IRTree model in which these two fundamentally different choices would be explained by the same latent variable is somewhat unnatural. For these reasons, we choose to utilize the MNRM family of models over the IRTree models in this paper.

In the multidimensional nominal response model, the probability of a participant endorsing an item category is calculated using equation (1)

(1) $$P\left(Y_{i}=k|\theta \right)=\frac{\exp\left({\tilde{\alpha }}_{ik}^{T}\theta +c_{ik}\right)}{\Sigma_{m=1}^{K}\;\exp\left({\tilde{\alpha }}_{im}^{T}\theta +c_{im}\right)},$$

where Y denotes the observed answer, K denotes the number of categories, ${\tilde{\alpha }}_{i}$ is a vector of slope parameters for item i , θ is the vector of participant scores on the dimension(s), and $c_{ik}$ is a category intercept. Subscript k refers to categories, and subscript i refers to items. Note that the first category intercept is constrained to zero to identify the model.

An adaptation of the multidimensional nominal response model presented in equation (1) is used in this study (Falk & Cai, 2016; Thissen & Cai, 2016). We chose this adaptation due to its flexibility in modelling various response styles. The use of a single, flexible model to generate data facilitates a straightforward test for the presence of different possible response styles, without introducing a confound by utilizing different model frameworks for the different response style conceptualizations. In addition, the model allows for a correlation between the substantive trait and the response style (or a correlation between response styles if multiple are present) to be modelled while simultaneously allowing for slopes to differ across items, in contrast to the model presented in equation (1). In the adaptation of the multidimensional nominal model, the probability of a participant endorsing an item category is calculated using equation (2) (Falk & Cai, 2016)

(2) $$P\left(Y_{i}=k|\theta \right)=\frac{\exp\left({\left[a_{i}⊙s_{k}\right]}^{T}\theta +c_{ik}\right)}{\Sigma_{m=1}^{K}\;\exp\left({\left[a_{i}⊙s_{m}\right]}^{T}\theta +c_{im}\right)},$$

where $a_{i}$ is a vector of slope parameters (1 for each dimension), $s_{k}$ is the *k*’th column of s , the matrix containing the scoring functions (1 row per dimension, 1 column per item), and ⊙ denotes Schur/Hadamard component-wise multiplication. Note that the ${\tilde{\alpha }}_{ik}^{T}$ vector of equation (1) has been split into an item-specific vector of slopes $a_{i}$ and a user-specified scoring matrix $s_{k}$ . This user-specified scoring matrix adds a great deal of flexibility to the model, since any response style or combination of response styles can be modelled using the scoring function matrix s . The scoring matrix s allows us to model ERS and MRS as either separate dimensions or as opposite poles of the same dimension.

To illustrate the scoring matrix, some examples are provided. For a model with no response style present (null model), five answer categories, and a single substantive dimension, the following scoring function can be utilized (Falk & Cai, 2016)

(3) $$[\begin{array}{lllll} 0 & 1 & 2 & 3 & 4] \end{array}.$$

Note that the multidimensional nominal response model given in equation (2) reduces to the generalized partial credit model (Muraki, 1997) when we use the scoring function defined in equation (3).

If one wants to model ERS and MRS as opposite poles of a single dimension (here labeled “ERS/MRS”), we could use the scoring functions

(4) $$\left[\begin{array}{lllll} 0 & 1 & 2 & 3 & 4\\ 2 & 1 & 0 & 1 & 2 \end{array}\right],$$

with the first row representing the scoring function of the substantive dimension (i.e., the dimension that relates to the latent trait of interest), and the second row representing the scoring function for the ERS/MRS dimension. With this scoring function, participants with a positive score on the second dimension will show a tendency towards the extreme categories and simultaneously show a tendency to avoid the middle category. Participants with a negative score on the second dimension will show the reverse pattern. Note that other values could be considered for the scoring function. The values in this study were chosen based on earlier research (Falk & Cai, 2016), and to ensure the gap between the extreme categories (1 and 5) and the adjacent categories (2 and 4) was of the same size as the gap between the middle category (3) and the adjacent categories (2 and 4).

Finally, if one wants to model ERS and MRS as two separate dimensions (here labeled ‘ERS + MRS’), one could use the scoring functions

(5) $$\left[\begin{array}{lllll} 0 & 1 & 2 & 3 & 4\\ 1 & 0 & 0 & 0 & 1\\ 0 & 0 & 1 & 0 & 0 \end{array}\right],$$

with the first row representing the scoring function for the content factor, the second row representing the scoring function for ERS, and the third row representing the scoring function of MRS (Falk & Cai, 2016). Due to each response style having its own row in the scoring function matrix, a tendency towards the extreme categories (positive score on the ERS dimension) does not necessarily entail a tendency to avoid the middle category (negative score on the MRS dimension), unlike the scoring function in equation (4).

## Methods

The present study was conducted using R (R Core Team, 2020), with code and data for the simulation provided at https://osf.io/gps9x/. To answer the research questions in this study, several conditions were examined. First, data were generated under a null model with no response styles present. Second, data were generated under a model with ERS and MRS as a single dimension. Finally, data were generated under a model with ERS and MRS as two separate dimensions. The conditions will be discussed in this order. Following the description of the conditions, we discuss how we analyzed the data and the outcome measures of the study.

### Null Condition

In this and all other conditions, five-option Likert-scale data were generated for participants according to the adaptation of the multidimensional nominal response model (Falk & Cai, 2016) in equation (2). To start, participant scores on the substantive trait were drawn from the standard normal distribution. The slopes $\alpha ∼Lognormal\left(0,0.3^{2}\right)$ to simulate a realistic range of item discriminations, while the item intercepts c were set to −2, −1, 0, −1, −2. These intercept values were chosen to ensure a symmetrical distribution of the answer probabilities (i.e., the distribution is mirrored, with for example the probability of endorsing category 1 with a trait score of −1 being equal to the probability of endorsing category 5 with a trait score of 1), where a participant has a maximal probability of choosing the middle category when they have a score of zero on the substantive trait of interest. Since we were interested in a model with no response styles present, we used the scoring function from equation (3). Category response probabilities for this set of item parameters can be found in Supplemental material A.

In the null condition, several factors were varied. First of all, the sample size and the test length are known to influence the accuracy of IRT model estimates (Akour & AL-Omari, 2013; Stone & Yumoto, 2004; Şahin & Anıl, 2017). Based on previous studies, we expected larger sample sizes and higher test lengths to lead to greater accuracy in distinguishing between models with no response styles, ERS/MRS or ERS + MRS (from here on referred to as “model classification accuracy”). Sample sizes of 250, 500, and 1000 were used, with test lengths of 10 and 20 items. Second, the number of substantive dimensions might influence the ease of detecting response styles such as ERS and MRS (Plieninger, 2017). We expected a higher number of substantive dimensions to lead to greater model classification accuracy. For this reason, data were generated with 1 and 2 substantive dimensions. Note that the substantive traits were independent of each other in conditions where two were modelled. We chose to model the substantive traits as independent of each other to maximize the difference of the conditions with two substantive traits to the conditions where only a single trait was present (which can be conceptualized as two traits with a correlation of 1 between them). When generating two substantive dimensions, the number of items per dimension was equal to the test length divided by two. For the null condition, the combination of all factors led to a total of 3 (sample size) *2 (test length) * 2 (number of substantive dimensions) =12 conditions.

### ERS/MRS Condition

The data-generating scoring functions for the ERS/MRS model are given in equation (4). The approach for this scenario is similar to the approach for the null model, except for the inclusion of a response style dimension. For the response style dimension, participants’ response style trait scores were drawn from a normal distribution with a mean of zero and a variable standard deviation. Here, the strength of the response style present is likely to affect results, with more extreme response styles being more easily detectable (Cho, 2013). In a search of the literature, little empirical information (and certainly no broad scientific consensus) emerged on what should be considered a weak, normal or strong ERS/MRS response style. We therefore chose to consider three values for the standard deviation of the response style: 0.6, 1, and 1.5. These values were specifically chosen in order to create an influence of a response style that is weak, medium, and strong, respectively, while still maintaining realistic category probabilities (considering the influences of the response style for being +1/-1 SD from the mean, while keeping the substantive dimension constant). Category response probabilities for these conditions can be found in Supplemental material A. We expected higher values of the response style standard deviation to lead to higher model classification accuracy. Because of the inclusion of the response style SD factor, this condition had 3 (sample size) ∗2 (test length) ∗ 2 (number of substantive dimensions) ∗3 (response style SD) =36 conditions.

### ERS + MRS Condition

The data-generating scoring functions for ERS and MRS being two separate dimensions are given in equation (5). This scenario differed from the ERS/MRS condition by modelling two response style dimensions instead of one. Note that when modelling two response styles simultaneously, an interesting aspect is the correlation between the slopes of these separate response styles. On the one hand, one might expect an item that is strongly affected by one response style to also be strongly affected by the other response style, implying a positive correlation between the response style slopes. On the other hand, one could also expect an item that evokes one response style to not necessarily evoke another response style, implying a correlation near zero between the response style slopes. When response style slopes are positively correlated, the model with ERS + MRS may be more similar to the ERS/MRS model, making it more challenging to correctly recover the data-generating model. To study the extent to which different values for this correlation might affect the classification accuracy, we thus examined both a scenario where the response style slopes are fully uncorrelated (correlation of 0) and a scenario where the response style slopes are identical (correlation of 1), a range of correlations we consider plausible. In the case of response style slope correlation values in between zero and one, we expect the classification accuracy values to fall somewhere in between the outcomes of these two extremes.

Response style trait scores were drawn from a bivariate normal distribution with a vector of zeros as the mean and a variable covariance matrix, which enabled a correlation between response styles to be specified. Note that the response style dimensions are always generated as independent from the substantive dimensions (i.e., the dimension(s) corresponding to the trait(s) of interest). The standard deviations of both response style dimensions were always equal to each other (i.e., both were weak, medium, or strong). The correlation between response styles was varied between −.5, 0, and .5. We expected a negative correlation between response styles to lead to a lower probability of the ERS + MRS response style being detected, since ERS + MRS response styles correlated at −1 are essentially identical to the ERS/MRS response style. Conversely, a positive correlation should lead to a higher probability of the ERS + MRS response style being detected. Addition of the response style correlation factor resulted in 3 (sample size) ∗2 (test length) ∗ 2 (number of substantive dimensions) ∗3 (response style SD) ∗3 (response style correlation) ∗2 (response style slope correlations) =216 conditions. To reduce the influence of sampling variability on the results, all conditions were replicated 250 times.

### Data Analysis and Outcomes

After generating the data, the mirt R package (Chalmers, 2012) was used to estimate the multidimensional nominal response model with each of the three scoring matrices described in Equations (3) to (5). The fitted model with the lowest AIC (Akaike, 1998) or BIC (Schwarz, 1978) was chosen as the preferred model for each respective criterion. AIC and BIC were utilized in this study for two reasons. First of all, AIC and BIC are both well-known model comparison indices that are frequently used in a wide array of settings. Second, the AIC is the most liberal (i.e., tends to prefer models with more parameters) model comparison index that is calculated by mirt, while the BIC is the most parsimonious (i.e., tends to prefer models with less parameters) model comparison index calculated by mirt. We thus chose these model comparison indices as they are least similar to each other and therefore provide most unique information.

Note that while the asymptotic performance of these two model fit indices is known (BIC will asymptotically select the true model if it is among the candidate models and assumptions are met, while AIC asymptotically minimizes loss functions regardless of the true model being a candidate), the same cannot be said for the performance of these indices for practically relevant finite sample sizes. To evaluate the performance of these two indices in finite sample size conditions, simulation studies are thus needed. An important note here is that even when the true model is under consideration (as in this simulation study), BIC may still perform worse than AIC in finite sample conditions (Vrieze, 2012). The present study will thus provide valuable information to researchers attempting to distinguish between ERS and MRS as a single dimension versus ERS and MRS as two distinct dimensions using the AIC and BIC in realistic, finite sample sizes.

The model with the lowest AIC/BIC matching the condition the data was generated in was considered a correct choice. The proportion of correct choices is referred to as “(model) classification accuracy” throughout the paper and is the outcome of interest. We are both interested in comparing the AIC and BIC to each other and examining the effect of the factors on model classification accuracy.

## Results

### Null Condition

In the null condition, the BIC achieved 100% classification accuracy across all conditions. We thus do not display a table for these results. As the AIC does not select the right model in all cases, its results are displayed in Table 1. Especially for low test length and small sample size, model classification accuracy decreases. While the overall classification accuracy of the AIC is not poor in and of itself, remaining above 80% in all cases, it is clear the BIC performs better in this condition. Overall, the BIC outperformed the AIC in the null condition by a decent margin (100% vs 92% correct).

**Table 1.** Percent of Cases Correct Model is Chosen When Using the AIC in the Null Condition

$\theta_{N}$	Number of items = 10			Number of items = 20		
	*N* = 250	*N* = 500	*N* = 1000	*N* = 250	*N* = 500	*N* = 1000
$\theta_{N}$ = 1	84	90	90	89	96	95
$\theta_{N}$ = 2	89	88	96	91	94	97

*Note.* Percentages are based on 250 observations per table cell. $\theta_{N}$ denotes the number of dimensions and *N* denotes the sample size.

To clarify the effects of the factors on the model classification accuracy, Table 2 displays the effects of the factors on the classification accuracy. Again, we do not show results for the BIC since 100% model classification accuracy was achieved in all conditions. For the AIC, increasing the sample size and test lengths leads to an increase in model classification accuracy. Increasing the number of substantive dimensions from 1 to 2 has little practical impact on model classification accuracy.

**Table 2.** Effects of the Factors on the Probability of the Correct Model Being Chosen for the Null Condition

Factors	Percent correct AIC
*N* = 250	88
*N* = 500	92
*N* = 1000	94
Number of items = 10	90
Number of items = 20	94
$\theta_{N}$ = 1	91
$\theta_{N}$ = 2	92

*Note.* N denotes the sample size and $\theta_{N}$ denotes the number of substantive dimensions. Percentages are based on 250 observations per table cell.

### ERS/MRS Condition

Tables 3 and 4 display the model classification accuracy for the BIC and AIC, respectively, in the ERS/MRS condition. Overall, the BIC and AIC perform very well here. Only for weak response styles in combination with a small sample size and short test length, accuracy drops below 95% for the BIC. The AIC somewhat outperforms the BIC in these specific conditions, but in general tends to reach a 100% classification accuracy less often than the BIC. In the ERS/MRS condition, the indices perform similarly overall (100% vs 99% correct, respectively).

**Table 3.** Percent of Cases Correct Model is Chosen When Using the BIC in the ERS/MRS Condition

Factors		Number of items = 10			Number of items = 20		
		*N* = 250	*N* = 500	*N* = 1000	*N* = 250	*N* = 500	*N* = 1000
$\sigma_{RS}$ = 0.6	$\theta_{N}$ = 1	91	100	100	100	100	100
	$\theta_{N}$ = 2	94	99	100	100	100	100
$\sigma_{RS}$ = 1	$\theta_{N}$ = 1	100	100	100	100	100	100
	$\theta_{N}$ = 2	100	100	100	100	100	100
$\sigma_{RS}$ = 1.5	$\theta_{N}$ = 1	100	100	100	100	100	100
	$\theta_{N}$ = 2	100	100	100	100	100	100

*Note.* N denotes the sample size, $\theta_{N}$ denotes the number of substantive dimensions, and $\sigma_{RS}$ denotes the response style standard deviation. Percentages are based on 250 observations per table cell.

**Table 4.** Percent of Cases Correct Model is Chosen When Using the AIC in the ERS/MRS Condition

Factors		Number of items = 10			Number of items = 20		
		*N* = 250	*N* = 500	*N* = 1000	*N* = 250	*N* = 500	*N* = 1000
$\sigma_{RS}$ = 0.6	$\theta_{N}$ = 1	96	98	100	99	100	100
	$\theta_{N}$ = 2	98	97	100	99	100	100
$\sigma_{RS}$ = 1	$\theta_{N}$ = 1	98	100	99	100	100	100
	$\theta_{N}$ = 2	99	98	99	100	99	100
$\sigma_{RS}$ = 1.5	$\theta_{N}$ = 1	99	99	100	100	100	100
	$\theta_{N}$ = 2	96	98	100	100	100	100

*Note.* N denotes the sample size, $\theta_{N}$ denotes the number of substantive dimensions, and $\sigma_{RS}$ denotes the response style standard deviation. Percentages are based on 250 observations per table cell.

Table 5 displays the effects of factors on model classification accuracy. As both model comparison indices show nearly perfect performance, we chose to not interpret effects in this condition in much detail. Instead, we limit our conclusion to the fact that increasing sample size and number of items increased performance of both the AIC and BIC somewhat. Increasing response style standard deviation improved the performance of the BIC, but not of the AIC. The number of substantive dimensions again has little to no impact on the results.

**Table 5.** Effects of the Factors on the Probability of the Correct Model Being Chosen for the ERS/MRS Condition

Factors	Percent correct BIC	Percent correct AIC
*N* = 250	99	99
*N* = 500	100	99
*N* = 1000	100	100
Number of items = 10	99	99
Number of items = 20	100	100
$\theta_{N}$ = 1	100	99
$\theta_{N}$ = 2	100	99
$\sigma_{RS}$ = 0.6	99	99
$\sigma_{RS}$ = 1	100	99
$\sigma_{RS}$ = 1.5	100	99

*Note.* N denotes the sample size, $\theta_{N}$ denotes the number of substantive dimensions, and $\sigma_{RS}$ denotes the response style standard deviation. Percentages are based on 250 observations per table cell.

### ERS + MRS Condition

For the conditions where data were generated using the ERS + MRS model, Tables 6 and 7 display the model classification accuracy for the BIC and AIC, respectively. Note that to facilitate comparison between the conditions where the response style slopes are correlated one versus zero, we report both classification accuracies separated by a “/” in each cell. Table 6 shows a very strong contrast between conditions for the BIC, with correct model choice varying between 0% and 100%. Conditions with low response style strength, negative response style correlations, low test lengths, and low sample sizes lead to especially poor performance. In general, the classification accuracy increases when the response style slope correlation is zero and decreases when the response style correlation is one. While the difference between the classification accuracies is usually not too large (i.e., less than 10%), it can make a substantial difference in some cases. For example, having a response style slope correlation of one instead of zero when the response style correlation is −.5, the response style standard deviation is 1, the number of substantive dimensions is 2, sample size is 1000 and the number of items is 10 results in a classification accuracy that is 36 percentage points higher. Overall, the results for the AIC in Table 7 seem to follow the same pattern as the BIC. The AIC does tend to be less variable in its model selection success, with percentage of correct choices varying between 20% and 100% rather than 0% and 100%. The AIC is clearly the better performer in the ERS + MRS condition, greatly outperforming the BIC (64/69% vs 92/94% correct, respectively).

**Table 6.** Percent of Cases Correct Model is Chosen When Using the BIC in the ERS + MRS Condition

Factors			Number of items = 10			Number of items = 20		
			*N* = 250	*N* = 500	*N* = 1000	*N* = 250	*N* = 500	*N* = 1000
$r_{RS}=-.5$	$\sigma_{RS}=0.6$	$\theta_{N}=1$	0/0	0/0	0/0	0/0	0/0	0/6
		$\theta_{N}=2$	0/0	0/0	0/1	0/0	0/0	0/6
	$\sigma_{RS}=1$	$\theta_{N}=1$	0/2	9/27	60/85	4/21	58/90	99/100
		$\theta_{N}=2$	1/2	7/20	46/82	5/21	48/82	99/100
	$\sigma_{RS}=1.5$	$\theta_{N}=1$	37/60	91/99	100/100	89/97	100/100	100/100
		$\theta_{N}=2$	32/54	80/96	100/100	90/99	100/100	100/100
$r_{RS}=0$	$\sigma_{RS}=0.6$	$\theta_{N}=1$	0/0	4/5	28/39	2/2	28/48	90/98
		$\theta_{N}=2$	0/0	2/2	22/33	2/3	23/36	86/97
	$\sigma_{RS}=1$	$\theta_{N}=1$	54/72	94/100	100/100	99/100	100/100	100/100
		$\theta_{N}=2$	45/61	93/99	100/100	96/100	100/100	100/100
	$\sigma_{RS}=1.5$	$\theta_{N}=1$	100/100	100/100	100/100	100/100	100/100	100/100
		$\theta_{N}=2$	100/100	100/100	100/100	100/100	100/100	100/100
$r_{RS}=.5$	$\sigma_{RS}=0.6$	$\theta_{N}=1$	1/1	16/18	66/82	10/11	75/91	100/100
		$\theta_{N}=2$	1/0	10/9	66/75	8/11	75/86	100/100
	$\sigma_{RS}=1$	$\theta_{N}=1$	92/97	100/100	100/100	100/100	100/100	100/100
		$\theta_{N}=2$	92/97	100/100	100/100	100/100	100/100	100/100
	$\sigma_{RS}=1.5$	$\theta_{N}=1$	100/100	100/100	100/100	100/100	100/100	100/100
		$\theta_{N}=2$	100/100	100/100	100/100	100/100	100/100	100/100

*Note.* N denotes the sample size, $\theta_{N}$ denotes the number of substantive dimensions, $\sigma_{RS}$ denotes the response style standard deviation, and $r_{RS}$ denotes the correlation between the response styles. To facilitate comparison between the conditions where the response style slopes are correlated one versus zero, respectively, we report both classification accuracies separated by a “/” in each cell. Percentages are based on 250 observations per table cell.

**Table 7.** Percent of Cases Correct Model is Chosen When Using the AIC in the ERS + MRS Condition

Factors			Number of items = 10			Number of items = 20		
			*N* = 250	*N* = 500	*N* = 1000	*N* = 250	*N* = 500	*N* = 1000
$r_{RS}=-.5$	$\sigma_{RS}=0.6$	$\theta_{N}=1$	21/31	33/49	52/78	23/47	63/88	94/100
		$\theta_{N}=2$	20/27	24/48	51/76	27/45	64/88	94/99
	$\sigma_{RS}=1$	$\theta_{N}=1$	70/84	96/99	100/100	98/100	100/100	100/100
		$\theta_{N}=2$	65/78	91/98	99/100	99/100	100/100	100/100
	$\sigma_{RS}=1.5$	$\theta_{N}=1$	98/100	100/100	100/100	100/100	100/100	100/100
		$\theta_{N}=2$	98/100	100/100	100/100	100/100	100/100	100/100
$r_{RS}=0$	$\sigma_{RS}=0.6$	$\theta_{N}=1$	57/62	90/92	98/100	89/97	100/100	100/100
		$\theta_{N}=2$	50/60	86/88	96/99	89/95	99/100	100/100
	$\sigma_{RS}=1$	$\theta_{N}=1$	100/100	100/100	100/100	100/100	100/100	100/100
		$\theta_{N}=2$	99/100	100/100	100/100	100/100	100/100	100/100
	$\sigma_{RS}=1.5$	$\theta_{N}=1$	100/100	100/100	100/100	100/100	100/100	100/100
		$\theta_{N}=2$	100/100	100/100	100/100	100/100	100/100	100/100
$r_{RS}=.5$	$\sigma_{RS}=0.6$	$\theta_{N}=1$	81/90	99/100	100/100	100/100	100/100	100/100
		$\theta_{N}=2$	82/84	97/99	100/100	100/100	100/100	100/100
	$\sigma_{RS}=1$	$\theta_{N}=1$	100/100	100/100	100/100	100/100	100/100	100/100
		$\theta_{N}=2$	100/100	100/100	100/100	100/100	100/100	100/100
	$\sigma_{RS}=1.5$	$\theta_{N}=1$	100/100	100/100	100/100	100/100	100/100	100/100
		$\theta_{N}=2$	100/100	100/100	100/100	100/100	100/100	100/100

*Note.* N denotes the sample size, $\theta_{N}$ denotes the number of substantive dimensions, $\sigma_{RS}$ denotes the response style standard deviation, and $r_{RS}$ denotes the correlation between the response styles. To facilitate comparison between the conditions where the response style slopes are correlated one versus zero respectively, we report both classification accuracies separated by a “/” in each cell. Percentages are based on 250 observations per table cell.

Table 8 displays the effects of the factors. Clear effects of factors are visible in the table. For both the AIC and BIC, increasing the sample size, the test length, the response style standard deviation, and the response style correlation leads to increased model classification accuracy. Decreasing the response style correlation leads to less accurate model selection, while the number of substantive dimensions again seems to have little impact on the results.

**Table 8.** Effects of the Factors on the Probability of the Correct Model Being Chosen for the ERS + MRS Condition

Factors	Percent correct BIC	Percent correct AIC
*N* = 250	49/53	85/89
*N* = 500	64/70	93/96
*N* = 1000	80/83	97/99
Number of items = 10	56/61	88/92
Number of items = 20	72/76	95/97
$\theta_{N}$ = 1	65/69	92/95
$\theta_{N}$ = 2	64/68	91/94
$\sigma_{RS}$ = 0.6	23/27	77/84
$\sigma_{RS}$ = 1	75/82	98/99
$\sigma_{RS}$ = 1.5	95/97	100/100
$r_{RS}$ = −.5	40/49	80/87
$r_{RS}$ = 0	76/79	97/98
$r_{RS}$ = .5	81/83	99/99

*Note.* N denotes the sample size, $\theta_{N}$ denotes the number of substantive dimensions, $\sigma_{RS}$ denotes the response style standard deviation, and $r_{RS}$ denotes the correlation between the response styles. To facilitate comparison between the conditions where the response style slopes are correlated 1 versus zero, we report both classification accuracies separated by a “/” in each cell. Percentages are based on 250 observations per table cell.

## Empirical Example

To further illustrate our approach, an empirical example is provided. In this empirical example, we first sought a dataset with a very large sample size, such that the response styles (if present) would be clearly distinguishable, and the correct model would likely be chosen based on our simulation results. To further study the performance of the two information criteria in real data, we subsequently used this empirical data set as the basis for an additional empirical resampling-based simulation study, intended to supplement the main simulation study covered in the previous section. By resampling empirical data, the complex structures present in real data are retained. Since we start with a very large dataset, and then use that as the basis for this simulation study, we can also be confident (due to the large sample size) in knowing which of the three models should actually be preferred for this setting. Additionally, we are still able to consider what the performance of the two information criteria would be if a more limited dataset with a smaller sample size or shorter test length had been considered.

To start, we obtained a dataset where response styles would easily be detectable if present. We chose to use public data provided by the Programme for International Student Assessment (PISA) for this purpose. In the 2022 PISA dataset (OECD, 2024), we opted to use two scales with ten five-category items each suitable for this example. The first scale measured assertiveness, with items such as “I take initiative working with my classmates,” and “I find it hard to influence people” (reverse coded). The second scale measured curiosity, with items such as “I like to know how things work” and “I am more curious than most people I know.” The response options were labeled “Strongly disagree,” “Disagree,” “Neither agree nor disagree,” “Agree,” and “Strongly agree” for both scales.

For this empirical example, we chose to examine the country with most participants to maximize expected classification accuracy. This resulted in us selecting the Spanish dataset with 28.925 participants, a sample size far above the sample size needed to reliably distinguish between the response style operationalizations based on results of our simulation study. Note that we chose the Spanish dataset merely for illustration and we make no claims regarding the generalizability of results obtained in this example.

As a first step, the GPCM, ERS/MRS, and ERS + MRS models were estimated using the mirt R package (Chalmers, 2012). For identification purposes, the means of all latent variables were fixed to zero and all variances were set to 1. All item slopes, intercepts, and covariances between the latent traits were freely estimated. Table 9 displays the mean items slopes and the estimated correlations between the traits for the different models. As can be seen in the table, the mean items slopes were slightly above 1 for both substantive dimensions under all models, matching the conditions in our simulation. For the ERS/MRS model, the mean item slope was slightly below 1, creating a similar strength of the response style dimension (i.e., a response style standard deviation of 1) as in the main paper. Finally, the ERS + MRS model found a mean ERS slope of 1.7 and a mean MRS slope of 0.7, indicating relatively strong ERS but weaker MRS. The correlation between the response style slopes was estimated to be .28, falling in between the two extremes examined in the simulation study.

**Table 9.** Mean items slopes and correlations between latent traits for the various models

Model	$\alpha_{\theta_{1}}$	$\alpha_{\theta_{2}}$	$\alpha_{RS_{1}}$	$\alpha_{RS_{2}}$	$r_{\theta_{1}\theta_{2}\;}$	$r_{\theta_{1}RS_{1}\;}$	$r_{\theta_{2}\;RS_{1}}$	$r_{\theta_{1}RS_{2}\;}$	$r_{\theta_{2}\;RS_{2}}$	$r_{RS_{1}RS_{2}}$
GPCM	1.2	1.1	-	-	.26	-	-	-	-	-
ERS/MRS	1.1	1.1	0.9	-	.21	−.02	.13	-	-	-
ERS + MRS	1.1	1.1	1.7	0.7	.21	−.05	.15	−.01	−.09	−.23

*Note*. ${\;\alpha }_{\theta_{1}}$ denotes the mean of the substantive slope for the first substantive dimension, $\alpha_{\theta_{2}}$ denotes the mean of the substantive slope for the second substantive dimension, $\alpha_{RS_{1}}$ denotes the mean of the first response style slope (ERS/MRS in the case of the ERS/MRS model, ERS in the case of the ERS + MRS model), $\alpha_{RS_{2}}$ denotes the mean of the second response style slope (MRS), and subscripts for the correlation r follow the same naming conventions.

The correlation between the substantive traits was estimated at .26 when no response styles were modelled. Adding either of the response style operationalizations to the model decreased the estimated correlation between the substantive traits to .21. Correlations between the substantive traits and the response styles were all below .2, while ERS and MRS were negatively correlated to each other (−.23).

To establish which of the fitted models was preferred in this dataset, the AIC and BIC were calculated for each of the three models. AIC values for the models in order of complexity were 739,588, 720,858, and 710,442, with the BIC values being 740,424, 721,875, and 711,649. That is, both the AIC and BIC pointed to the ERS + MRS model as the preferred model.

In addition to a dataset where the response styles would be clearly distinguishable, we aimed to create several conditions where the response styles would potentially be more difficult to distinguish, while remaining fairly certain about which of the three models should actually be preferred. This was achieved by sampling people and items (all without replacement to match the structure of the real dataset) from the aforementioned Spanish dataset. Conditions where the response styles would be more difficult to detect were created based on the conditions in the simulation study. As a first factor, we varied the number of scales/dimensions between 2 (both curiosity and assertiveness) and 1 (only assertiveness). We varied sample size by sampling a number of people (250, 500, or 1000) and varied the number of items by selecting a certain number of items (10 in the case of a single scale, 10 or 20 in the case of 2 scales). In the condition with 10 items from 2 scales, we sampled items without replacement. Taking all factors into account, we obtain 9 conditions where the performance of the two information criteria could be examined. In each condition, we randomly sampled people and items 100 times to reduce the influence of sampling variability.

After sampling people and items, we once again calculated the AIC and BIC for the null, ERS/MRS, and ERS + MRS models and established the preferred model. In this case, we assumed the ERS + MRS model was the true data-generating model, since it was preferred in the larger sample where no items, scales, or people were cut. A classification other than the ERS + MRS model was thus counted as a “wrong” classification. Note that to ensure convergence of the models, it was required that every item category was endorsed at least once. If this was not the case after the random sampling, a single missing value (the first missing value for the item in question) was replaced with the non-endorsed item category (e.g., if no participants answered a 1 on a given question, one missing value was replaced with a 1). Table 10 presents the results for all 9 conditions.

**Table 10.** Model Classification Accuracy for the Two Information Criteria

Factors		Number of items = 10			Number of items = 20		
		*N* = 250	*N* = 500	*N* = 1000	*N* = 250	*N* = 500	*N* = 1000
BIC	$\theta_{N}=1$	18	85	100	-	-	-
	$\theta_{N}=2$	1	22	91	25	98	100
AIC	$\theta_{N}=1$	100	100	100	-	-	-
	$\theta_{N}=2$	90	100	100	100	100	100

*Note.* N denotes the sample size, $\theta_{N}$ denotes the number of substantive dimensions. Percentages are based on 250 observations per table cell.

In general, the results of this empirical simulation study match the results of the earlier simulation study in the paper when the response style standard deviation is 1, taking into account a somewhat stronger detectability than when the response style correlation is −.5 and the response style slope correlation is one, but a weaker detectability than when the response style correlation is 0 and the response style slope correlation is zero due to the empirical example falling in between these conditions. The AIC generally outperformed the BIC, which was to be expected given the more complicated data-generating model and the artificially created conditions where the response style would be difficult to detect. The importance of choosing the right information criterion to detect which response style is present, especially when sample size and test length are low, is reemphasized.

## Discussion

The present study set out to establish whether it is possible to empirically distinguish between extreme responding and midpoint responding as separate dimensions or opposite points of a single dimension in a given dataset using the AIC and BIC, and if so, under which conditions and with what degree of model classification accuracy this is possible. Data were generated under three general conditions to answer this question. These conditions will be discussed below in order of appearance.

In the null condition, the absence of a response style in the data could be established well by both the AIC and BIC. The sample size and test length had a positive influence on model selection accuracy, but only for the AIC. The BIC performed perfectly in this condition, while the AIC obtained a respectable overall correct model classification. The BIC is thus the better model comparison index to establish the absence of response styles in data. This is an expected result, as the BIC more readily selects more parsimonious models, and the null model is the most parsimonious model of the three possible models considered.

Both the BIC and the AIC showed excellent performance in detecting the presence of ERS/MRS in a dataset under nearly all conditions. The AIC outperformed the BIC when the response style SD was low, while the reverse was true when the response style SD was medium or high. Both model comparison indices were overall well-suited for detecting this particular response style, with neither index outperforming the other consistently. The effects of factors were minor in this condition.

The condition with ERS and MRS as two separate dimensions was the place where both the difference between the model comparison indices and the effects of the factors were most pronounced. For negative response style correlations and weak to medium response style strengths, the BIC was almost completely unable to correctly detect the model that generated the data in the considered conditions, instead often erroneously preferring the simpler null **or** ERS/MRS model. Even for the zero or positive response style correlations, the performance of the BIC was very poor in the presence of a weak response style.

The AIC’s performance generally followed the same trend as the BIC, but its overall performance was substantially higher. Both model comparison indices were strongly affected by the sample size, the test length, the response style standard deviation and the response style correlation for correct model classification. Of particular note in this condition was the effect of the response style slope correlation on the detectability of the ERS + MRS response styles, with a correlation of zero leading to a higher detectability than a correlation of one. The number of substantive dimensions hardly affected the results in this or other conditions. The absence of a detectible effect of the number of substantive dimensions could be due to the fact that the total number of items was kept constant, thus halving the number of items per substantive dimension when two were present. The gain in information due to having two separate substantive dimensions rather than one substantive dimension may thus have been offset by a loss of information per substantive dimension.

Overall, the AIC was most definitely the better model comparison index for the purpose of correctly detecting the presence of two separate response styles, outperforming the BIC. Note that this result holds despite the true model being among the candidate models, indicating that the asymptotic performance of the BIC in selecting the true model is not close to being reached in the sample sizes examined in this study. Thus, the preference of the BIC for more parsimonious models may prevent it from correctly detecting the presence of ERS and MRS as two separate response styles in many realistic application settings. This finding also held for the real data used in the empirical example, where the AIC greatly outperformed the BIC in a dataset where ERS + MRS were present, particularly when sample sizes were low.

Several limitations are present in this study. As a first limitation, conducting a simulation requires various simplifying assumptions to be made. These assumptions are unlikely to hold in practice and may influence the results obtained. For example, the item slopes and intercepts were the same for all items, which will not occur with a real questionnaire. In addition, all participant trait and response style scores were drawn from a normal distribution with a mean of zero and a standard deviation of one. In practice, it is likely participants will not all draw from the same distribution, especially if groups differ in age, gender, race, and other predictors of response styles. The values chosen in this study for the response style standard deviation, while based on careful consideration of item category curves and the resulting item score distributions for different values of the latent variables, are still arbitrary in a sense, and different values could potentially have been used. Furthermore, it could be the case in practice that the true model is not among the candidate models. Future research should examine these scenarios. Additionally, the response styles were set to affect each item equally, which is also unlikely to hold in practice. Since midpoint responding has been found to be affected by participant fatigue, later items may for example be more affected by it than earlier items. Finally, only two response styles were modelled in this study, while more may be present in real-life data. The addition of other response styles to a dataset might complicate the process of detecting which response styles exactly are present. Future research can expand on this study by experimenting with different participant trait score distributions, varying item parameters, varying operationalizations of response styles, considering what happens if the true model is not among the candidate models, varying effects of response styles on items, adding other response styles (e.g., investigating acquiescent and disacquiescent response style as two separate points of a single dimension or two separate dimensions), and other variations on this study design. Including the correlation between the substantive trait and the response style trait(s) as a design factor may be of particular interest, as it could be conceived that this may complicate the detection of responses styles (since less unique information is present) or instead aid in the detection of response styles (since information can be “borrowed” between correlated traits).

As a second limitation, the present study focused on comparing the AIC and BIC in terms of model classification accuracy. While these are among the most commonly used and most readily available model selection criteria, it is possible other indices are better suited to this task. Future research should look into the performance of other model comparison indices.

From the findings of this study, several recommendations for practical research arise. First of all, the use of the AIC or BIC seems to make quite a difference in some conditions when attempting to detect response styles. Which index is best to use will thus depend on research aims and hypotheses. If a researcher wants to check whether response styles may be present but wants to avoid false positives (i.e., incorrectly concluding that they are present), use of the BIC is the safest choice, since it is more conservative in terms of moving away from the null model in favor of one of the response style models, while still having reasonable power to select a model with response styles in it if they are indeed present. In contrast, if a researcher already expects a response style to be present but wishes to distinguish between ERS/MRS or ERS + MRS, the AIC performs notably better and may be preferred.

Another approach that may be considered is a combined approach, where first the BIC is used to establish the possible presence of a form of ERS and MRS, and if the BIC indicates to move away from the null model, subsequently the AIC is used to establish whether they should be modelled using a single or two separate dimensions. Results of this combined approach are presented in Supplemental Material B and appear promising. Based on the results in this supplementary material, both the use of the AIC alone or the combination of the AIC and BIC is defensible. The AIC performs a bit better than the combined approach in the ERS + MRS condition, but the combined approach is better at detecting the absence of any response style in the data, due to the BIC being conservative with respect to moving away from the null model. Which approach is used must thus be determined by weighing the cost of detecting a response style that is not present against the cost of not detecting a response style that is present. Using the BIC alone is not recommended, as this results in inferior or equal performance in every condition compared to the combined approach.

Besides the choice between the AIC and BIC, the role of factors must also be discussed. For selecting the right model, the sample size and the test length must be sufficient, especially for the more complex multidimensional response style models. While the response style standard deviation and the response style correlation are not changeable by the researcher, they must nevertheless not be neglected. One important note is that in a condition with a negative response style correlation and a weak to moderate response style, both the AIC and BIC were frequently not able to recover the data-generating model even when test length and sample size increased. Researchers should take note that in conditions where response styles are negatively correlated and/or the response style standard deviation is low, sample sizes and test lengths may need to be increased even further to correctly select the data-generating model than one would expect otherwise. Researchers may make use of the estimated parameters of the two response style models to gain insight into which of the considered conditions is most similar to theirs. That is, a researcher could estimate the ERS + MRS model (regardless of whether it is selected over the ERS/MRS model or not) and consider the results from Tables 6 and 7 for the conditions most similar to the estimates that were obtained. Design factors of the simulation with a large impact would be of particular interest here. If those results suggest that one is unlikely to select the ERS + MRS model even if it is correct, it would be reasonable to conclude that there is insufficient evidence for distinguishing between the two model options.

Overall, the question of whether it is possible to distinguish between ERS and MRS as being a single versus two separate response styles in realistic practical settings can be answered with a yes. Only when a negative response style correlation occurs for the ERS + MRS condition in combination with a low response style standard deviation do we really run into problems distinguishing the two versions that cannot straightforwardly be solved by simply increasing the test length or the sample size within reasonable limits. A researcher utilizing a test with 20 items and around 500 participants should thus not anticipate major problems in the vast majority of cases when attempting to distinguish ERS/MRS from ERS + MRS. If a sample size of 500 participants is not possible, even sample sizes of 250 often suffice for this goal. It should again be noted that we do not recommend using a BIC-only approach here, as this can lead to poorer performance even under the conditions outlined above.

## Supplemental Material

Supplemental Material - Distinguishing Between Models for Extreme and Midpoint Response Styles as Opposite Poles of a Single Dimension versus Two Separate Dimensions: A Simulation Study Supplemental Material for Distinguishing Between Models for Extreme and Midpoint Response Styles as Opposite Poles of a Single Dimension versus Two Separate Dimensions: A Simulation Study by Martijn Schoenmakers, Maria Bolsinova, and Jesper Tijmstra in Applied Psychological Measurement

## Data Availability

All data and code used in the study can be found via the link https://osf.io/gps9x/.
