# Supplementary material for: Distinguishing Between Models for Extreme and Midpoint Response Styles as Opposite Poles of a Single Dimension versus Two Separate Dimensions: A Simulation Study
Source: Appl Psychol Meas. 2025 Sep 13;50(3):78–97. doi: 10.1177/01466216251379471 (PMC12433433; doi:10.1177/01466216251379471)
Supplement: Supplementary file 1 [file sj-pdf-1-apm-10.1177_01466216251379471.pdf]

Supplementary Material A

Category Response Probabilities For an ERS/MRS Dimension Being Present

Figure A1:

Category Probabilities when ERS is Zero

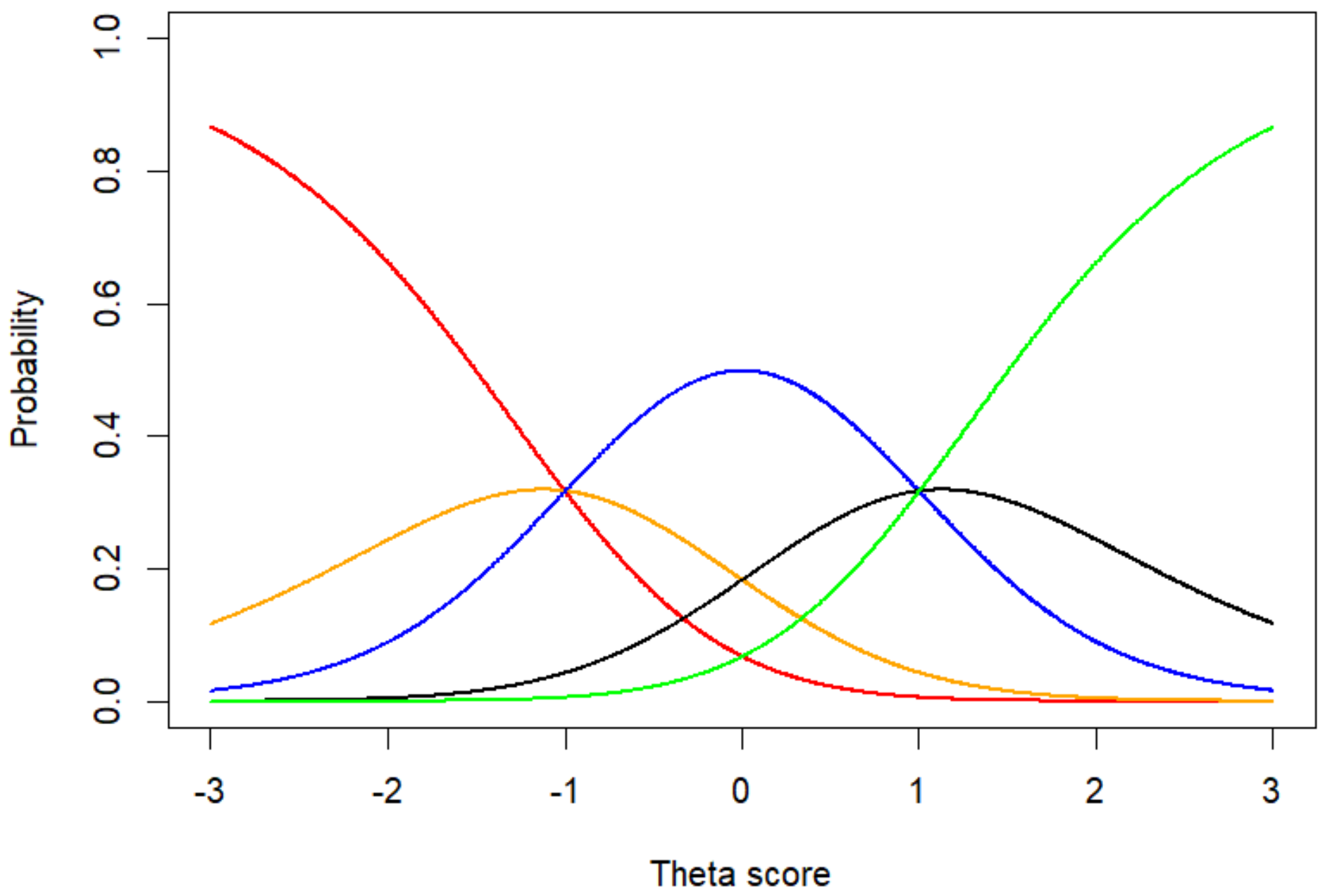

Note. Red marks category 1, orange marks category 2, blue marks category 3, black marks category 4 and green marks category 5.

Figure A2:

Category Probabilities when ERS is 0.6 (top left), 1 (top middle), 1.5 (top right), -0.6 (bottom left), -1 (bottom middle) or -1.5 (bottom right)

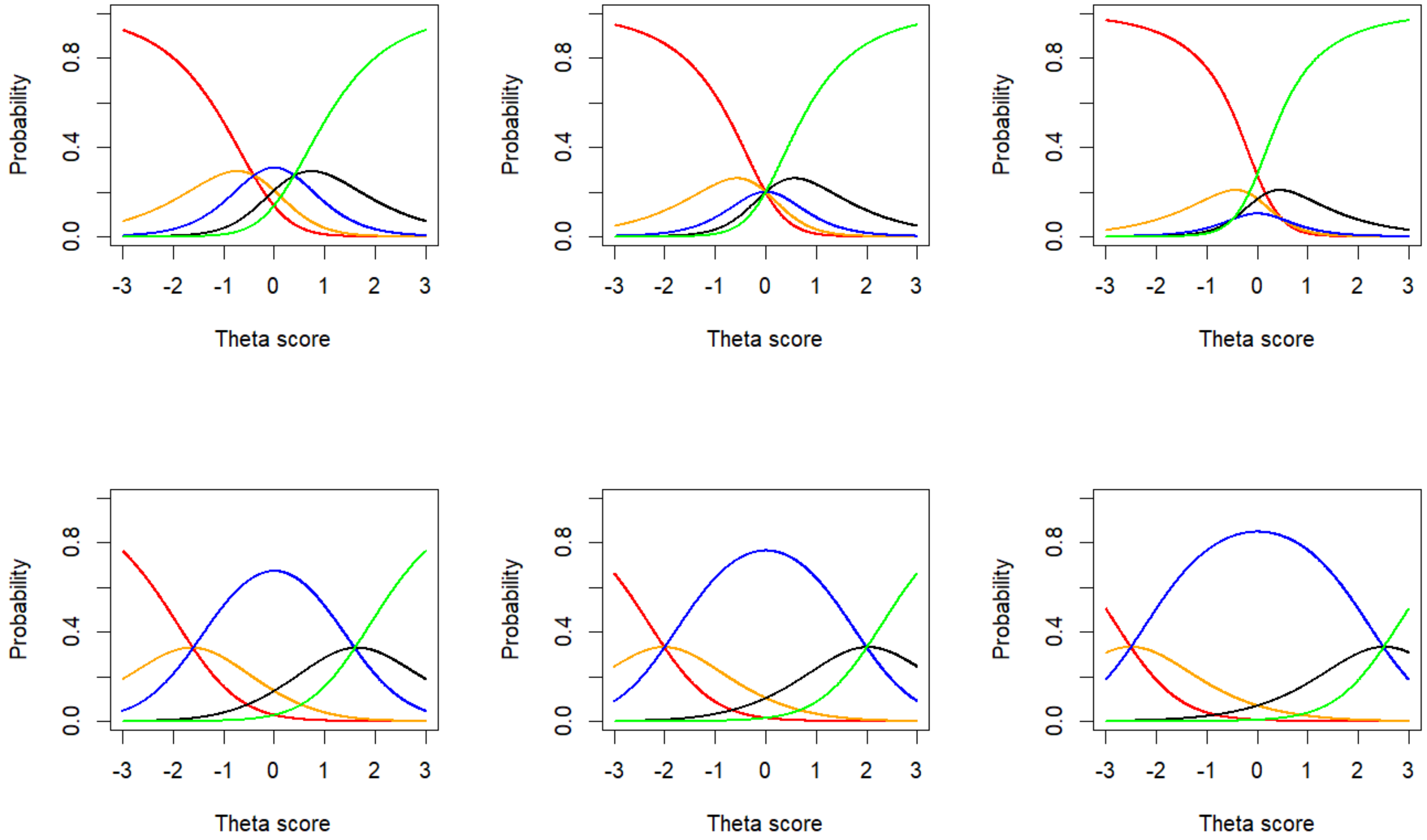

Note. Red marks category 1, orange marks category 2, blue marks category 3, black marks category 4 and green marks category

Supplementary Material B

Results if BIC is used to establish presence of response styles, and AIC to establish which response style is present

Since the BIC (and thus also the combined approach) show perfect performance in the control conditions, this table is not shown here. Instead, Table B1 shows the performance of the combined BIC/AIC approach in the ERS/MRS condition. In Table B1, we see a slight decline in performance compared to both the AIC and BIC. The combined approach thus performs worse than both approaches individually in this condition. It must be noted that this decrease in performance only occurs in a few cells and is not very large. The overall performance using this approach is 99% compared to ~100% for the BIC and 99% for the AIC.

Table B1

Percent of Cases the Correct Model is Chosen When Using the BIC to Establish the Presence of a Response Style, then the AIC to Establish Which Response Style is Present in the ERS/MRS Condition

Factors		Number of items = 10			Number of items = 20		
		N = 250	N = 500	N = 1000	N = 250	N = 500	N = 1000
σ_{RS} = 0.6	θ_N = 1	88	97	100	99	100	100
	θ_N = 2	92	96	100	99	100	100
σ_{RS} = 1	θ_N = 1	98	100	99	100	100	100
	θ_N = 2	99	98	99	100	99	100
σ_{RS} = 1.5	θ_N = 1	99	99	100	100	100	100
	θ_N = 2	96	98	100	100	100	100

Note. N denotes the sample size, θ_N denotes the number of substantive dimensions and σ_{RS} denotes the response style standard deviation. Percentages are based on **250** observations per table cell.

Table B2 shows the results for the ERS + MRS condition. Here, the performance of the AIC/BIC combined approach is in-between that of the BIC and AIC by themselves. Especially for the low sample size, the low response style strength, and the low test length, performance decreases compared to the AIC-only approach. The approach does yield better results than the BIC only approach. Overall, this combined approach leads to 84/87% overall correct model classification, compared to 64/69% for the BIC and 92/94% for the AIC. If we somewhat arbitrarily assume that the probability of being in the null condition, ERS/MRS condition, or ERS + MRS condition are equal ($\frac{1}{3}$), we would thus arrive at the following model classification accuracies:

BIC only: $1 * \frac{1}{3} + 1 * \frac{1}{3} + .675 * \frac{1}{3} = .89$

AIC only: $0.92 * \frac{1}{3} + .99 * \frac{1}{3} + .93 * \frac{1}{3} = .95$

AIC/BIC combined: $1 * \frac{1}{3} + .99 * \frac{1}{3} + .865 * \frac{1}{3} = .95$

Table B2

Percent of Cases the Correct Model is Chosen When Using the BIC to Establish the Presence of a Response Style, then the AIC to Establish Which Response Style is Present in the ERS + MRS Condition

Factors	Number of items = 10	Number of items = 20

			N = 250	N = 500	N = 1000	N = 250	N = 500	N = 1000
$r_{RS} = -.5$	$\sigma_{RS} = 0.6$	$\theta_N = 1$	14/21	33/48	52/78	23/47	63/88	94/100
		$\theta_N = 2$	14/16	24/46	51/76	26/45	64/88	94/99
	$\sigma_{RS} = 1$	$\theta_N = 1$	70/84	96/99	100/100	98/100	100/100	100/100
		$\theta_N = 2$	65/78	91/98	99/100	99/100	100/100	100/100
	$\sigma_{RS} = 1.5$	$\theta_N = 1$	98/100	100/100	100/100	100/100	100/100	100/100
		$\theta_N = 2$	98/100	100/100	100/100	100/100	100/100	100/100
$r_{RS} = 0$	$\sigma_{RS} = 0.6$	$\theta_N = 1$	16/10	65/62	96/98	67/64	100/99	100/100
		$\theta_N = 2$	14/11	61/54	92/98	66/58	97/99	100/100
	$\sigma_{RS} = 1$	$\theta_N = 1$	100/100	100/100	100/100	100/100	100/100	100/100
		$\theta_N = 2$	91/100	100/100	100/100	100/100	100/100	100/100
	$\sigma_{RS} = 1.5$	$\theta_N = 1$	100/100	100/100	100/100	100/100	100/100	100/100
		$\theta_N = 2$	100/100	100/100	100/100	100/100	100/100	100/100
$r_{RS} = .5$	$\sigma_{RS} = 0.6$	$\theta_N = 1$	1/2	18/20	66/82	11/12	75/91	100/100
		$\theta_N = 2$	2/0	10/10	67/75	8/12	75/86	100/100
	$\sigma_{RS} = 1$	$\theta_N = 1$	92/97	100/100	100/100	100/100	100/100	100/100
		$\theta_N = 2$	94/98	100/100	100/100	100/100	100/100	100/100
	$\sigma_{RS} = 1.5$	$\theta_N = 1$	100/100	100/100	100/100	100/100	100/100	100/100
		$\theta_N = 2$	100/100	100/100	100/100	100/100	100/100	100/100

Note. N denotes the sample size, θ_N denotes the number of substantive dimensions, σ_{RS} denotes the response style standard deviation, and r_{RS} denotes the correlation between the response styles. To facilitate comparison between the conditions where the response style slopes are correlated 1 versus zero, we report both classification accuracies separated by a / in each cell. Percentages are based on 250 observations per table cell.

From these results, it is clear both the AIC and the combined approach are defensible. The conservative researcher will prefer the combined approach, and the liberal researcher will prefer the AIC. Alternatively, one could take a more Bayesian approach and shift around their prior beliefs about the response style condition they are in to obtain the index best suited to them, with a heavier weight on the null condition resulting in a preference for the combined approach, and a heavier weight on the ERS + MRS condition resulting in a preference for the AIC. This use of priors must naturally be well reasoned and justified. The BIC only approach leads to almost identical or severely inferior performance to the AIC/BIC combined approach in all conditions, and we would thus recommend against this approach for the conditions studied in this paper.
